# From the Eye of the Storm: An IoT Ecosystem Made of Sensors, Smartphones and UAVs †

**DOI:** 10.3390/s18113814

**Published:** 2018-11-07

**Authors:** Milan Erdelj, Borey Uk, David Konam, Enrico Natalizio

**Affiliations:** 1 Cognitech DOO, Sombor 25000, Serbia; milan.erdelj@cognitech.rs; 2 Syntony GNSS, Toulouse 31000, France; borey.uk@syntony.fr; 3 Thales SIX GTS, Gennevilliers 92230, France; david.konam@thalesgroup.com; 4 Université de Lorraine, Centre national de la recherche scientifique (CNRS), Loria, Campus Scientifique, Nancy 54506, France

**Keywords:** Unmanned Aerial Vehicles, mobile crowdsensing, cyber-physical systems, disaster management

## Abstract

The development of Unmanned Aerial Vehicles (UAV) along with the ubiquity of Internet of Things (IoT) enables the creation of systems that, leveraging 5G enhancements, can provide real-time multimedia communications and data streaming. However, the usage of the UAVs introduces new constraints, such as unstable network communications and security pitfalls. In this work, the experience of implementing a system architecture for data and multimedia transmission using a multi-UAV system is presented. The system aims at creating an IoT ecosystem to bridge UAVs and other types of devices, such as smartphones and sensors, while coping with the fallback in an unstable communication environment. Furthermore, this work proposes a detailed description of a system architecture designed for remote drone fleet control. The proposed system provides an efficient, reliable and secure system for multi-UAV remote control that will offer the on-demand usage of available sensors, smartphones and unmanned vehicle infrastructure.

## 1. Introduction

Many efforts are being made to recognize and forecast the occurrence of a natural disaster, to react in an efficient manner to the disaster when they occur, and to quickly and efficiently assess the damage, fix and restore normal state [1,2,3,4].

Large-scale natural disasters test the most fundamental human instinct of survival by inflicting massive, and often unpredictable, loss to life and property. Various types of natural disasters have been classified according to the technology that can be used to respond to them [5]: geophysical (earthquake, tsunami, volcano, landslide, and avalanche), hydrological (flash-floods, debris flow, and floods), climatological (extreme temperature, drought, and wildfire) and meteorological (tropical storm, hurricane, sandstorm, and heavy rainfall), among others, have caused the loss of many lives in addition to increase in material losses in the order of 100–150% over the last 30 years [6]. Acknowledging the need for bolstering disaster resilience, this paper contributes a vision of leveraging the latest advances in wireless sensor network (WSN) technology, unmanned aerial vehicles (UAVs) and mobile crowdsensing to enhance the ability of network-assisted disaster prediction, assessment and response.

Around 47% of the overall losses and 45% of the insured losses derived from inland flooding that occurred in Europe, Canada, Asia and Australia. Altogether, at around US$ 45bn, losses from natural catastrophes were below the average amount for the past ten years (US$ 85bn). Insured losses totaled approximately US$ 13bn. Thus, in this paper, we focus our attention on inland flooding events.

Some recent works tackle the problem of a system design dedicated to use in natural disasters. Usually, the device-to-device communication network systems are applicable to many different wireless technologies, notably using smartphones for relaying emergency messages [7]. In some cases, the parts of the cellular network infrastructure that survived the natural disaster can play a vital role in transmitting the information by forming a multi-hop communication link [8]; in other cases, terrestrial vehicles can support or request UAVs to cooperate to offer connectivity [9].

The novelty of our work in respect to the existing literature is that we provide a novel architecture, composed of three main elements: sensors, UAVs and crowdsensing devices. Furthermore, we offer an extensive technical description of the whole system as well as of each of its components, and provide an in-depth overview of technologies used for data encoding or video-streaming. In previous works on the subject, Luo et al. presented a similar system architecture which enables data processing in the cloud [10]. However, the description of the components provides a high-level overview, whereas in our work the technology stack of the system is completely detailed and makes use of production-approved open-source technology. Furthermore, in Ref. [10], smartphones are not considered as a part of the control and monitoring interface for the operators. Smartphone connectivity has been considered in Ref. [11], where the authors proposes using UAVs to establish a link with the device of a missing person, after a disaster event happened. In this case, the authors provided some significant insights into the communication technologies and realized a demo to show the correct functioning of their architecture. However, no characterization of the real and perceived information flows among the different components is given, and the smartphone, included in the architecture, only has the function of sending a help message. In our architecture, smartphones are integrated by following the new paradigm of crowdsensing communications. The crowdsensing component is also absent from the Paparazzi system [12], which permits remote control of multiple UAVs by the ground station through classic communications technologies. Furthermore, the usage of the MAVLink protocol for UAV communications is considered as a future extension, whereas our architecture is based on MAVLink protocol, which makes it flexible and easy to manage and extend. None of the mentioned works present such an extended validation and performance evaluation campaign as our work.

This work describes the communication architecture for a system of systems (SoS) composed of UAV, smartphones, and sensors to transmit telemetry and data streaming, which we proposed in the framework of the project IMATISSE (Inundation Monitoring and Alarm Technology In a System of SystEms). This work extends our previous contribution in Ref. [13], and its main contributions are the following:

1. the proposal of a SoS architecture for disaster management;
2. the definition of roles and functions for each SoS component of the proposed architecture;
3. the definition and characterization of the real and perceived information flows among the different components; and
4. the practical implementation and validation of the proposed architecture.

The outline of this paper is the following: components of the proposed architecture are presented in Section 2, operating modes in Section 3, functional architecture in Section 4, and its implementation in Section 5. The evaluation of video streaming and data architecture performance is provided in Section 6. Conclusions are drawn in Section 7.

## 2. Architecture Components

**Figure 1:**
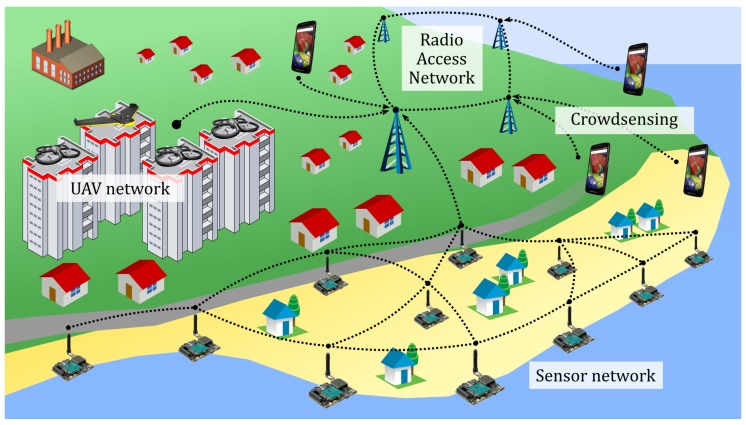
Overall IMATISSE architecture.

**Figure 2:**
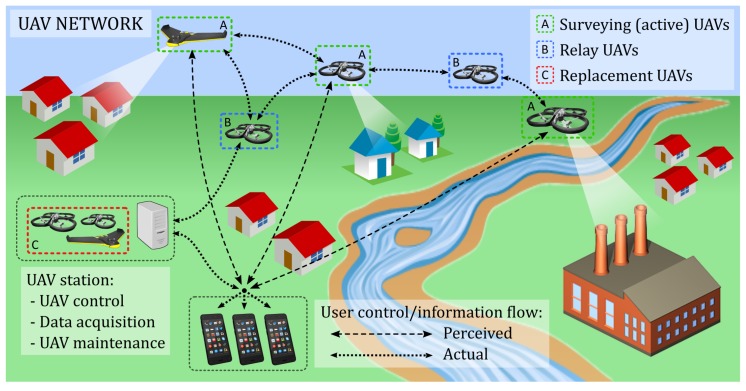
UAV network used for surveillance and communication support.

**Figure 3:**
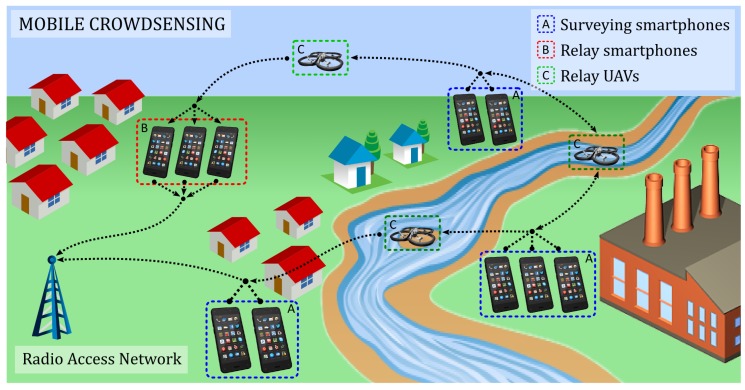
Mobile crowdsensing.

The system of systems we propose for forecasting, assessing and responding to an inland flooding, comprises three main components, as shown in Figure 1:

- A sensor network, assumed to be composed of cheap and widely available nodes, has three roles in this context:
  – Monitoring the river/stream by acquiring information, the data are fed to a web server that stores the data, creates the statistics and triggers the alarms based on the collected information;
  – Monitoring bed and bank of the river, where it acts as a danger trigger; and
  – support for communication, as it connects other system components (UAVs and smartphones). The WSN, in this case, can connect isolated smartphones that cannot access the Radio Access Network (RAN) and inform the web server about their location and status.
- A UAV network that, through a UAV server, allows a 24/7 vision on distance of the places hit by the flooding and a virtual UAV service that offers a continuous connection between the users and the UAVs. The dedicated UAV server should be equipped with a set of UAVs, the power generator, and an operator that takes care of the drones, stores them in the UAV server and operates its movement (Figure 2).
- Smartphones that people use on a daily basis constitute the third component of the system. The idea is to develop a smartphone application that could achieve the following:
  – allow people’s crowdsensing to voluntarily trigger the rescue alarm, followed by the creation of an endangered area map useful to rescue teams (Figure 3);
  – communicate with the WSN to get the individual sensor readings; and
  – connect directly to the UAV network to transmit its status information (notably position) towards the rescue teams and get the information regarding the emergency procedure, escape plans, etc.

Two more components of the proposed architecture, the web server and the UAV server, which, respectively, link the different components of the system together and manage the UAVs, are not represented in Figure 1 for sake of simplicity.

## 3. Operating Modes

The IMATISSE system works according to two operating modes, normal operating mode and emergency mode, which reflect the disaster alert/preparedness phase and the first response and assessment phase, respectively.

### 3.1. Normal Operating Mode

In the normal operating mode, only the WSN represents the active part of the system. The WSN executes the task of acquiring the data regarding the river flow and level, forwarding the information towards the web server. Although smartphones are being used by their owners, the application dedicated to crowdsensing in the context of this project is launched only in the case of an emergency. In the case an increase of the water level is detected by the WSN or the emergency application is launched by a smartphone user, the system goes into emergency mode.

### 3.2. Emergency Mode

After the WSN or the smartphone application has triggered the alarm, the web server activates the UAV server. The emergency responders can request the connection to a UAV through the UAV server that grants the users’ request with the 24/7 available real-time vision-on-distance service. The emergency responders are referred to as main users in the rest of the paper to distinguish them from the regular users that are the smartphone owners involved in the disaster management through the crowdsensing application. The 24/7 availability of the service is provided by the autonomous system comprising multiple UAVs that are automatically replaced when the battery level of the active UAV reaches a critical threshold [14]. The replacement is being done by running the maintenance algorithm on three sides: active UAV, replacement UAV and the UAV server.

Both WSN and smartphone application can act as triggers, providing the web server (and thus the main user) with the essential information about the scope and the localization of the emergency. In the case where WSN could not perform its task of triggering the alarm on time, the smartphone application triggers the system. Emitting its location and status information, the smartphone application provides a potential focal point upon which the UAV subsystem should act. Gathering the information from different points in the network, the web server creates an emergency map, together with automated escape paths for stranded people. The information is transmitted towards the smartphone owners in both affected and endangered areas. In this way, smartphone owners can trigger the system, but also use the same application as a warning and useful information receiver.

The majority of information processing is done within the web server, where all information regarding the app users, their activity, river level, forecast, etc., is stored as well. Video and image processing gathered by UAVs could be performed on the actual UAVs, by implementing video recognition and analysis algorithms.

### 3.3. Interconnection of System Components

**Figure 4:**
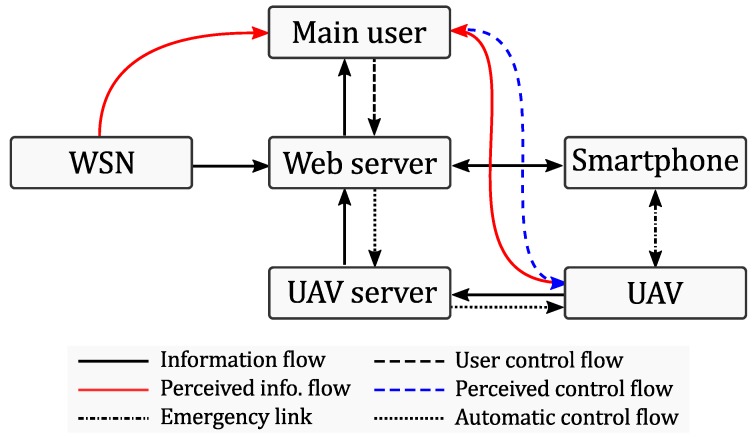
Interconnection among the system components.

Figure 4 shows the interconnection of the three system components: sensors, UAVs, smartphones through the UAV server and the web server.

The UAVs are gathered by fleets, where each fleet of UAVs sends telemetry data, while each UAV sends its video. All the data sent by the UAVs are received by the UAV server, which is connected to the UAVs through a local wireless network. In return, the UAVs receive Mavlink commands from the UAV server.

The UAV server centralizes all the data sent by the UAVs. Additionally, it also exposes each video stream for the web clients and also receives the command messages sent by the web server.

The Web server is the central element of the architecture: it stores telemetry and stream processing data into a database, and also provides an API to the web clients. The core of the web server is a program written in Golang which is responsible for launching the different modules in several threads.

The web server comprises the following modules:

- API: The API is responsible for exposing a RESTful API to the clients and enables two-way communication between the clients and the UAVs. On the one hand, the web application can query the database through the API module to retrieve data such as the last telemetry of a UAV or retrieve a snapshot of the video stream. On the other hand, clients, if authorized, can also send commands to the UAV fleet.
- Processing modules: These modules are responsible for processing data coming from the UAV fleet. Each type of data is assigned to a specific sub-module:
  – Streaming: Manages the websocket stream video servers and synchronizes the different video inputs (UAV streams) with the outputs (video players which are requesting a given stream).
  – Screenshot: Manages the reception of the snapshot resulting from the video stream and its analysis by OpenCV (by using face-detection algorithms) as well as allows the recording of streaming information into the database (addresses where UAVs publish their streams, and the addresses where the web client can retrieve them).
  – Telemetry: Processes telemetry data received and stores them in a database.

## 4. Functional Architecture

### 4.1. Overview

**Figure 5:**
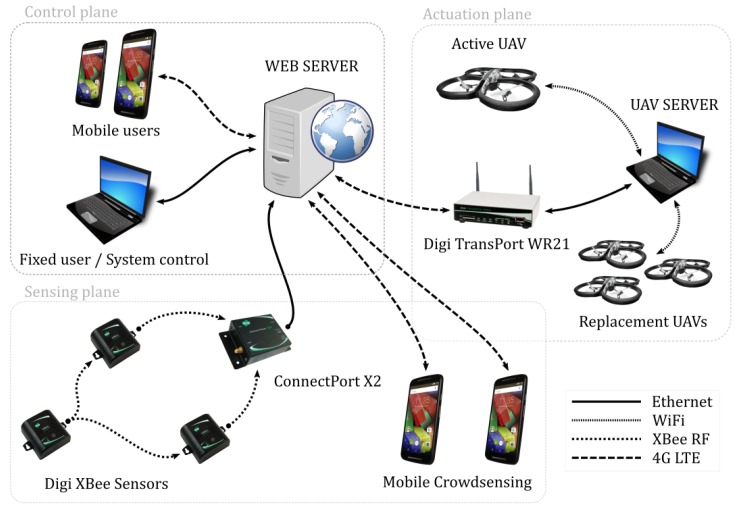
Functional description of the proposed architecture.

This section describes the function planes and the hardware needed to implement them (Figure 5). The central component of the system is the web server, which receives, sends and stores all the sensed data, user commands, acquired video stream from the UAVs and alerts generated by the mobile devices through the crowd sensing paradigm.

**Sensing plane.** The overall system of systems developed in this project relies on the readings from the WSN that focuses on river monitoring. The WSN monitors the water level near the river banks as well as around important infrastructure, thus performing the basic infrastructural monitoring as well. If the water level exceeds a certain predefined threshold, an alarm signal is transmitted towards the web server that can analyze and forward the alarm to other components of the system.

Sensor nodes that are used in the project are Digi XBee enabled ready-to-use sensors, which come in plastic casings, are battery powered and integrate acceleration, temperature, luminosity and humidity sensors. The communication modules integrated in these sensors is the Digi 868 RF protocol that operates on 868 MHz and that is characterized by 1–4 km of communication range with up to 80 kbps data rate. Data emitted by the sensors are collected at the sensor gateway (Digi ConnectPort X2) and transmitted, in normal operating mode, towards the web server through a 5G dedicated link.

The second part of the sensing plane comprises the smartphones used by the people that happen to be around the disaster area. By using the pre-installed mobile emergency application, they have the possibility to participate in disaster alarm and sensing by sharing their view on the disaster. The application installed on the smartphones allows regular users to report the incident, and send the incident report together with its exact location to the web server.

**Control plane.** The control plane represents the part of the system that is dedicated to the main users of the system: the emergency responders, who can use fixed or portable computer as well as mobile phones to connect to the central component of the system, the web server. The web server should have the capabilities of storing and analyzing the data that are fed by the WSN and displaying this information in human readable form. Besides the information sent by the sensor network, it should also integrate the information acquired by the crowdsensing platform. Mobile devices provide the platform with the location and information on events happening around the places of interest that the emergency responder should be able to consult and extract. If the need for the actuation is recognized, the responder sends a request for a UAV to move to the hit area. The available UAV then establishes the connection and video stream towards the emergency responder, who should have the complete control over it.

**Actuation plane.** After the responder’s request has been received, the UAV server activates the UAVs, and establishes the video stream towards the user via the web server. All video, video analysis and control messages are transferred by the UAV server. The goal of the UAV server is to overview the set of available drones and to observe the state of the active drones, besides transmitting all the information to and from the web server. The replacement drones are also under control of the UAV server in order to provide the uninterrupted service offered to the user. In the moment of replacement, the most important point is the handoff of the control signals and the video stream from the active to the replacement drone. After the handoff has been done, the replacement drone becomes the active one and the previously active drone returns back to the charging station to recharge its battery.

### 4.2. Sensor Integration and Visualization

The WSN is implemented using the available battery-powered wireless sensors with integrated capability of acceleration, temperature, humidity and light sensing. All the individual sensors in the network are programmed to periodically transmit their sensor readings and forward them towards the collection point—a WSN gateway that is connected to the dedicated web server for information storage.

Sensors that are used for this particular application are Digi XBee LHT sensors with integrated XBee 868 MHz communication modules. Their sampling frequency and message routing are easily configured using the dedicated GUI. Additional water presence/level sensor should be added on one of the available analog pins on the sensor board. The acquired data are transmitted towards the WSN gateway Digi ConnectPort X2. The setup of the gateway is done in a Python script, with the goal to assemble all the sensor readings, format them and transfer them towards the web server through the Ethernet port.

**Figure 6:**
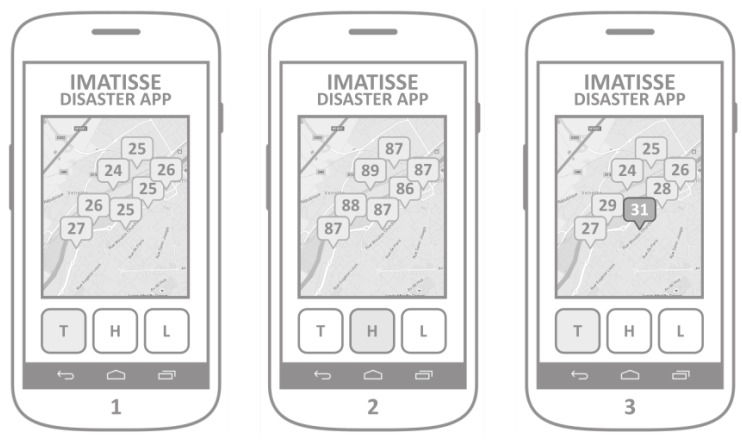
WSN visualization application.

The main goal of the implemented WSN visualization application is to show the measurements provided by the WSN. The Digi XBee LHT sensors are equipped with a temperature, humidity and ambient light sensor modules, which are configured to measure these values periodically and send them towards the collection point (Digi XBee sensor gateway). When the application is launched, it retrieves all the sensors readings from the web server. The map integration allows the user to see the geographical distribution of the measurements. Three buttons, T, H, and L, represent the particular physical value that is being displayed (temperature, humidity, and ambient light; Figure 6).

Therefore, when a user presses the T button, the application would display the geographical distribution of temperature readings (Screen 1), or the humidity readings when the button H is pressed (Screen 2). The simple web server implementation includes the verification of measure values to attach the warning field to the retrieved measurement. This warning field is used by the application in order to display a warning message and the value of the measurement that surpassed the predefined threshold. Values that are out of the normal value range are displayed together with other values, but in a different color or shape (Screen 3).

### 4.3. UAV Network Integration

**Figure 7:**
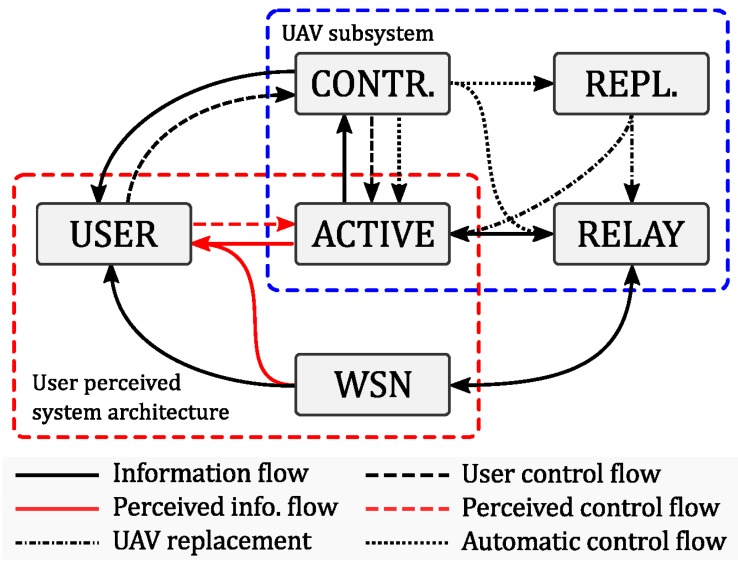
Architecture of the UAV server.

The architecture of a mobile UAV server with the goal of controlling the UAV network is presented in Figure 7. In conjunction to the server, a specialized application dedicated to the control of a fleet of Unmanned Aerial Vehicles (UAVs) is developed as well.

Usually, the control of a fleet heavily relies on controlling individual UAVs, where each UAV has its own dedicated operator. That solution introduces an important scalability issue in the case where a network of UAVs needs to be used. The approach described in this article relies on the implementation of advanced UAV deployment algorithms based on real-time neighborhood discovery and virtual force deployment. In this case, a fleet of autonomous UAVs needs only a common target to optimally deploy, without the need for individual operators. When the target has been defined and transmitted to the fleet, UAVs autonomously deploy and cover the target, while offering their video streams to the fleet controller (the user of the application). The controller can then connect to any of the individual video streams, while dynamically changing the position and viewpoint of the target. The application provides the simultaneous snapshot ability, i.e., provides the possibility to gather the camera snapshots from all the UAVs in the fleet in the same moment. These instantaneous snapshots from different angles are then uploaded to the web server, and used for the 3D point cloud construction. The application sends its requests towards the dedicated UAV server, and therefore does not directly control the individual UAVs but only provides the common target to the fleet as a whole.

**Figure 8:**
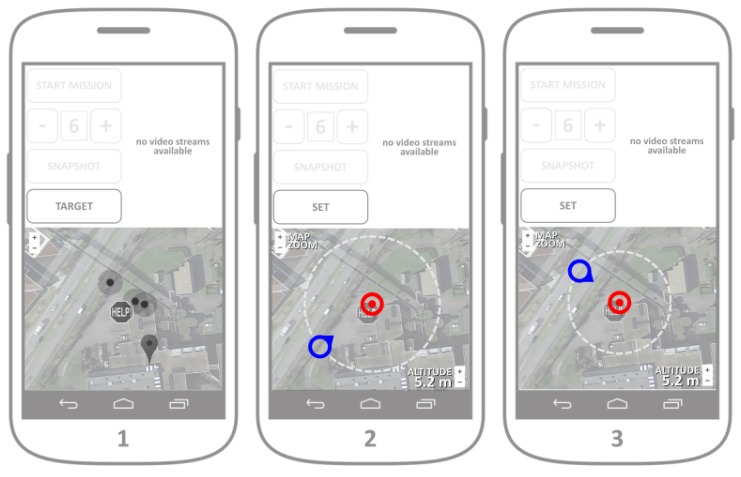
Fleet control application.

The application comprises two visual segments (Figure 8):

- Control/streaming area (upper part of the screen): The controls and the video streaming are displayed in this segment.
- Map area (lower part): This segment is used for the graphical representation of the real-time fleet deployment.

The fleet control application is related to the crowdsensing application (Section 4.4) with the same capability of connecting to the web server in the initialization phase, and displays the reported accident map in the map area of the application (Figure 8, mockup of Screen 1).

**Figure 9:**
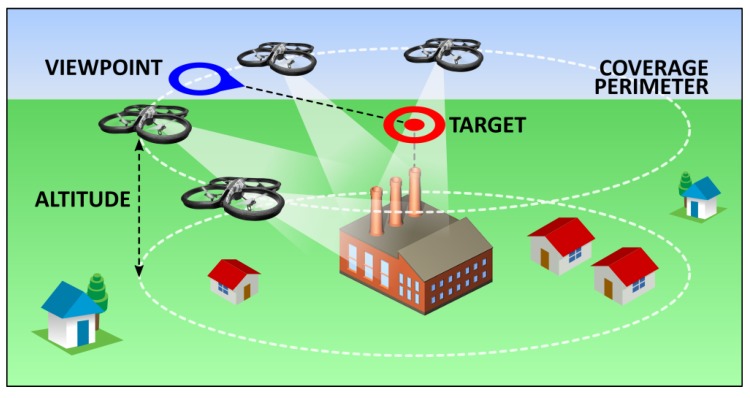
Fleet control illustration.

After analyzing the accident map displayed in the map segment of the main screen, the operator should proceed by setting up the fleet target by pressing the TARGET button. Without the target being initially set up, all other command buttons should be disabled by default. By pressing the TARGET button, the main user proceeds to setting up the target position, viewpoint and the coverage perimeter (mockup of Screens 2 and 3). Target setup is essential for the control of a fleet as a whole, since the deployment algorithm installed in each individual UAV relies on the common target position, the desired viewpoint and the coverage perimeter (distance from each individual UAV towards and the common target). The formation around the target position is inspired by the work in Ref. [15]. The meaning of each of these terms is illustrated in Figure 9.

**Figure 10:**
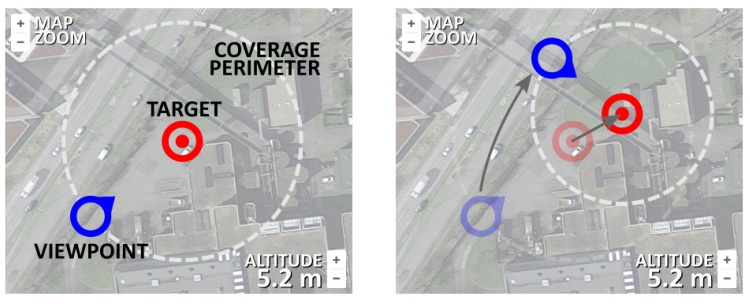
Fleet target setup.

The target setup (including the target position, viewpoint and perimeter) is easily executed by changing the position of the two position markers representing the target position and the viewpoint (Figure 10). Their mutual distance represents the coverage radius that defines the coverage perimeter. Map zoom is provided by default after the maps integration. When the operator is satisfied with the target placement, all information regarding the target is sent towards the UAV server after the button SET is pressed (Figure 8, mockups of Screens 2 and 3).

**Figure 11:**
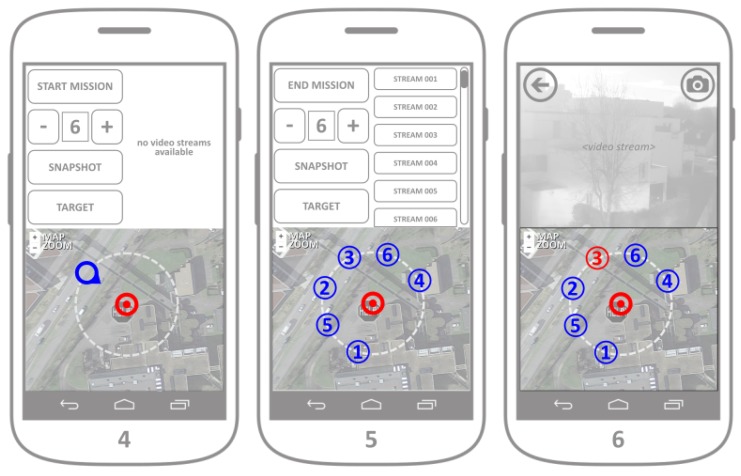
Fleet control application.

After the target has been set following the previous steps, the user has the option to start the mission by pressing the button START MISSION. The default fleet of UAVs assumes the use of six autonomous UAVs, however their number can be changed by pressing the buttons + and −, which informs the UAV server about the desired number of UAVs in the fleet, and displays the current number of UAVs on the screen (Figure 11, mockup of Screen 4). After the mission has started and the UAVs in the fleet have been deployed to their desired positions (defined by the target, viewpoint and coverage perimeter), their received individual geolocations are displayed in the map segment. Besides reporting its real-time geolocation, each individual UAV opens a video streaming channel, that a responder can connect to. Each individual streaming ID is directly related to the UAV IDs displayed in the map segment during the fleet deployment (Figure 11 mockup of Screen 5). A responder can visualize a video streaming both by clicking to a video stream channel offered in the control/streaming segment of the application, or by pressing any of the UAV markers in the map segment. After the video stream is chosen either by clicking on one of the stream buttons or on an individual UAV marker, the chosen video stream is displayed in the control/streaming segment of the application (Figure 8 mockup of Screen 6). By pressing the button in the upper left corner of the streaming segment, responders can close the video streaming and get back to the application main screen.

While the video stream is being displayed, the responder can change the position of the target by clicking on the target marker. A press on the target marker hides the UAV markers and displays the viewpoint market, thus allowing the change of target parameters even when the fleet is airborne. After the responder is satisfied with the target change, another press on the target marker hides the viewpoint marker and displays back the UAV markers (and their ID numbers). In both the control and streaming segments of the application screen, the responder has the option of synchronized camera snapshot (by pressing the SNAPSHOT button on the control, or the camera button on the streaming screen). Synchronized snapshot ability allows the responder to gather instantaneous photo collection from all the UAVs in the fleet. This set of snapshots is transmitted and stored on the web server, for the later use in order to generate a 3D point cloud of the covered target. The mission can be ended by clicking on the END MISSION button in the control segment. After the button has been clicked, a request for mission termination is sent towards the UAV server, which guides the fleet towards their collection point.

### 4.4. Crowdsensing Integration

The crowdsensing application allows smartphone owners to easily report the accident (via appropriate user interface) to the dedicated web server, so that the map of the accident can be created and shared with other users. This application also supports the visualization of the accident and flood occurrence distribution, as well as the alarm in the case the user happens to be in close proximity to the previously reported accident or a flood occurrence. The application allows the user to send a urgent help request that dispatches UAVs to its geographical location.

**Figure 12:**
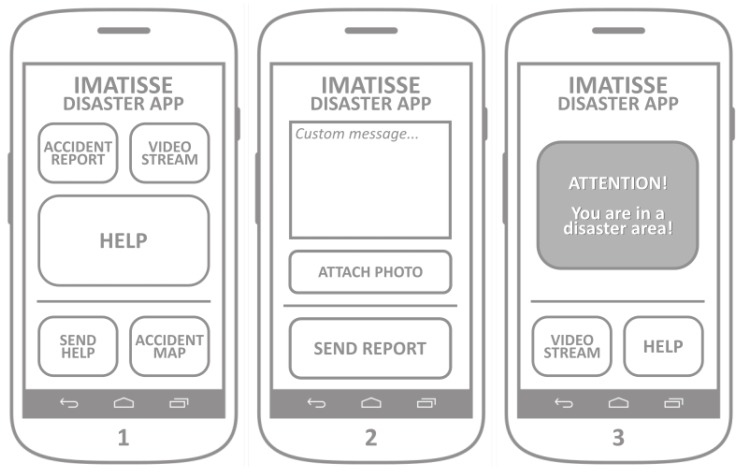
Crowdsensing application.

The mockup of the application screens is presented in Figure 12. The main screen (Figure 12, Screen 1) allows the user to easily report the accident by pressing the ACCIDENT_REPORT button. After the button is pressed, the application gathers the device’s localization information and shows Screen 2. By pressing the button SEND_REPORT, the application sends the accident report message to the web server. The accident report message sent to the server includes a set of basic predefined accident properties (e.g., perimeter, duration, etc). Optionally, a short text message and/or a photo of the accident can be added and sent together with the accident report. Running a background process, the application should periodically poll the server for changes in the list of accidents, which is used for the accident alarm and the accident map. After checking the device’s location, if the device is located within the perimeter of any of the accidents, the application launches the alarm, as shown in Screen 3. The alarm screen offers the VIDEO_STREAM and HELP buttons, with the same functionality of the buttons from the main screen.

During the application initialization, it connects to the main server to retrieve the list of previously registered accidents.

**Figure 13:**
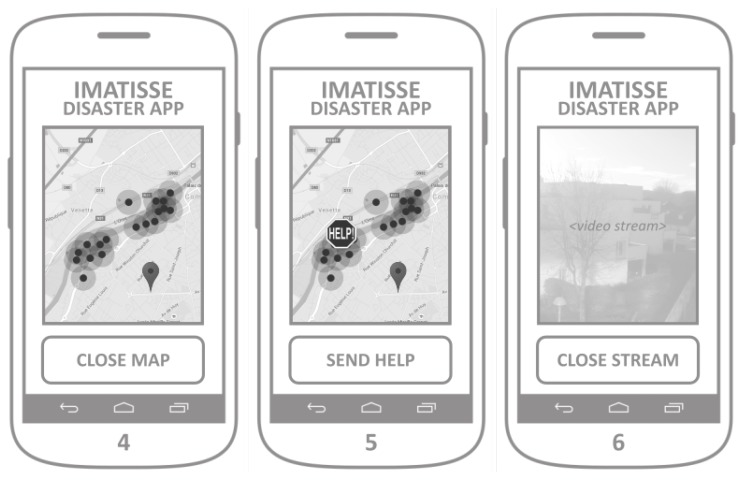
Crowd sensing application.

Clicking on the button ACCIDENT_MAP launches application Screen 4 (Figure 13), where the geographical distribution of reported accidents is presented with the maps integration.

Button HELP gets the device’s location and sends two messages to the server: (1) accident report with current location; and (2) the drone request with its current location. It is intended to be used in the emergency situations where an application user needs help and requests a drone to be dispatched to its location. The button SEND_HELP makes use of the same maps integration screen developed for the ACCIDENT_MAP functionality, but extends it with a possibility to pin a location on the map. It launches Screen 5, where a user can pin the desired location, which allows him to report an accident on a different location than its own, and to dispatch a drone on the desired location. The button SEND_HELP on Screen 5 sends the same two messages as by pressing the HELP button on the main screen, the difference is in the geographical location sent to the web server. Advanced functionality of the application represents the possibility of a real-time video streaming. By clicking on the button VIDEO_STREAM, the phones camera is activated, the application shows Screen 6, it establishes a video streaming channel and it registers it on the web server.

### 4.5. Web Server

**Figure 14:**
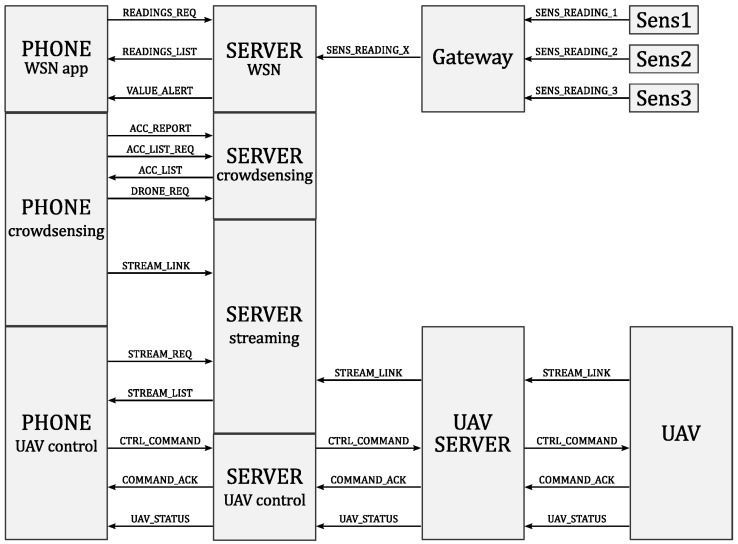
Web server structure.

The web server contains a database with all the information about users, their activity logs, the information about available and replacement drones, as well as the information provided by the WSN. Furthermore, the server keeps all the information about the availability of UAV servers and active UAVs (available UAVs on each server). The server script allows the transmission of control messages from the responders and transmission of video stream from the active drone back towards the responder. The exchange of the web server with the different components of the proposed architecture is illustrated in Figure 14.

## 5. Architecture Implementation

This section first introduces the technologies chosen for implementing the software of IMATISSE into a proof of concept system. Then, it presents the role they have into the proposed architecture. Finally, it introduces the encoding/decoding algorithms that play an important role in providing low latency and high quality multimedia transmission.

### 5.1. Chosen Technologies

It is worth noting that the technologies chosen to implement the proof of concept system have been selected after an accurate study of the existing technologies to use those that would allow a smooth integration in a larger 5G IoT ecosystem. The technologies chosen for the proof of concept system are the following:

- Communication library: ZeroMQ (http://zeromq.org);
- Data transmission format: Flatbuffers (https://google.github.io/flatbuffers/);
- Database and data visualization: ElasticSearch (http://elasticsearch.org) and Kibana (https://www.elastic.co/products/kibana);
- Programming languages: NodeJS (https://nodejs.org) and Golang (https://golang.org);
- Image processing library: OpenCV (https://opencv.org); and
- Video encoding/decoding library: FFmpeg (https://www.ffmpeg.org) and JSMpeg (http://jsmpeg.com).

ZeroMQ is a high-performance asynchronous messaging library, aimed at use in distributed or concurrent applications. It provides a message queue, but, unlike message-oriented middleware, a ZeroMQ system can run without a dedicated message broker.

FlatBuffers is an efficient cross platform serialization library and it was originally created by Google for performing critical applications. What makes FlatBuffers special is that it represents hierarchical data in a flat binary buffer, in such a way that it can still be accessed directly without parsing and unpacking, while supporting data structure evolution. FlatBuffers require only small amounts of generated code, and just a single small header as the minimum dependency, which is very easy to integrate. According to benchmarks, it is lighter than JSON.

ElasticSearch is a distributed search engine based on Apache Lucene. It has become one of the most popular search engines, and is commonly used for log analytics, full-text search, and operational intelligence cases. When coupled with Kibana, a visualization tool, ElasticSearch can be used to provide real time analytics using large volumes of log data. ElasticSearch offers REST API, a simple HTTP interface, and uses schema-free JSON documents making it easy to index, search, and query data.

FFmpeg is a multimedia framework, able to decode, encode, transcode, multiplex and stream multimedia flows. It supports the most obscure ancient formats up to the cutting edge. It is also highly portable—FFmpeg compiles and runs under a wide variety of build environments, machine architectures, and configurations.

JSMpeg is a video player written in JavaScript that consists of MPEG-TS demuxer, MPEG1 video and MP2 audio decoders, WebGL and Canvas2D renderers and WebAudio sound output. JSMpeg can load static videos via Ajax and allows low latency streaming via web sockets. It can work in any modern browser (Chrome, Firefox, Safari, and Edge). JSMpeg can connect to a web socket server that sends out binary MPEG-TS data. When streaming, JSMpeg tries keeping latency as low as possible—it immediately decodes everything it has, ignoring video and audio timestamps altogether.

OpenCV (Open Source Computer Vision) is a library of programming functions mainly aimed at real-time computer vision for all operations related to image processing. The library is composed of around 3000 algorithms, including sets of both classic and cutting edge computer vision and machine learning algorithms. These algorithms can be used to detect and recognize faces, identify objects, classify human actions in videos, track camera movements, track moving objects, and extract 3D models of objects. The architecture proposed in this paper relies on this technology due to its richness in the algorithms it offers.

As described in Ref. [16], there are three ways of communicating between software blocks, using a database, services, or messages.

In our architecture, we use the following three methods:

- Communication through a service is used between the API module and the clients.
- Communication through a database is used between the API module and the Processing submodules.
- All other communication uses messages with ZeroMQ.

Encoding protocols is also described in depth in Ref. [16]. In this part, we explain which data encoding technologies we chose for each block, by describing their general usage and their use in our implementation.

**Figure 15:**
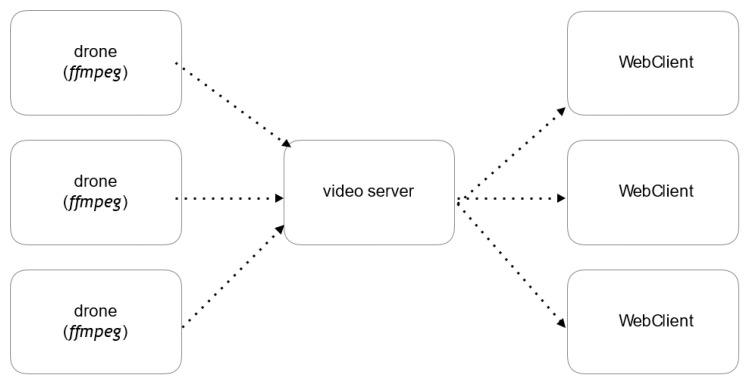
Multimedia transmission architecture.

The system implements a multimedia server in NodeJS that offers an access point available to UAVs allowing them to send their video streams, and an access point to allow web clients to retrieve the stream. With this method using the publisher/subscriber pattern, the server automatically manages the different UAVs in a completely independent and transparent way (Figure 15).

### 5.2. Comments on Fault Tolerance

The autonomy of UAVs in the system facilitates two aspects of fault tolerance:

- Error confinement with the isolation of the suspected faulty node preserves the system reliability.
- System readjustment gives nodes adaptability capacities that ensure the continuation of the service in the case of loss of some nodes.

As the components of the system must operate autonomously, the software architecture must show a high level of decomposition for the nodes to have well identified services. For example, the UAV emitter only takes care of sending information and video streams while the screenshot unit deals only with snapshot and image processing.

Security-wise, the *Mavlink* protocol offers a 12-round RC5-based message encryption. Such encryption is considered efficient for text up to $2^{44}$-bit length. Mavlink security issues are tackled extensively in Refs. [17,18]. FlatBuffers messages are binary-encoded but not encrypted by default. Thus, we need to encrypt messages with a symmetric key, which can encrypt itself with an asymmetric key. RSA encryption is recommended for its reliability.

### 5.3. Comments on the UAV Usage

Several practical implementation issues arise in the case of the combined usage of UAVs with sensor and smartphone network. One of the most evident issues is the energy consumption and the autonomy of a UAV. Average commercial UAVs achieve the autonomy of about 10–20 min of airborne operation, depending on the computational load and the weight of the payload a UAV carries. Taking into account the energy consumption issues, we propose the implementation of the automatic handover algorithm for continuous operation of UAVs [14].

Another important issue is the possible UAV collisions or failures during the operation in a fleet. This issue is usually resolved by implementing a fleet formation algorithm that includes the repulsive virtual forces among the neighboring UAVs. Taking into account that each UAV executes a periodic broadcast of its location and status in the network, all the neighboring UAVs that receive the information can calculate the repulsive movement forces that would avoid the collision with each other. Furthermore, a minimal safety distance among the UAVs needs to be defined prior to the UAV fleet deployment. To address the failure issues and keep the fleet functional, we advocate the usage of distributed multi-robot deployment algorithms [19] that can automatically reconfigure the fleet even with multiple simultaneous UAV failures.

## 6. Performance Evaluation

In this section, we perform video streaming and telemetry data transmission performance evaluation, for the cases with and without simulated network disruptions.

### 6.1. Video Streaming Performance

This section provides measurements of the streaming quality of our prototype in its conditions of use. As far as the streaming latency is concerned, these tests can help determine the difference of clock with respect to the display produced by video streaming.

For video player management, we tested two configurations: (1) video players are in separate web clients; and (2) video players are inside the same web page. Each configuration is tested with {1, 2, 3, 4, 5, 10} clients. Table 1 shows the results for Configuration 1, while Table 2 shows the results for Configuration 2. The latencies in both tables are expressed in seconds.

We observe fairly similar results for both configurations. Indeed, for both, the latency is not perceptible until the first five viewers. However, for many video players, the latency is much more noticeable in Configuration 2. We are convinced this is due to the JavaScript nature of the video player and the web page obviously has more work in its background process. Note that these tests were made with a video resolution of 640 × 480 px but some tests with video resolution of 1280 × 960 px showed similar results.

Furthermore, a disadvantage of JSMpeg comes from its use of canvas element. While the web page is not displayed by the browser, the page content is not rendered (even though background tasks such as video retrieving continue to progress). It is only when we come back to the web page that the content of the canvas is changed, which produces an extra latency by displaying in canvas all previous stored data.

### 6.2. Data Architecture Performance

As we are bound by resource constraints and our intent is to smoothly integrate our architecture in a 5G IoT context, which will make massive use of virtualization techniques, we started virtualizing the software architecture. Indeed, we used a virtual test environment to assert the proper functioning of the architecture. The first thing we had to do is to virtualize each part of the architecture into a virtual machine. Instead of using virtual machines, we used containers with *Docker* (https://www.docker.com).

In place of virtualizing the entire OS, the kernel is shared across containers, thus reducing the load on the host machine. This enables us to start a high number of containers with a reduced resource consumption compared to an equivalent number of virtual machines.

The following step is to setup a virtual network for the containers. We create and manage the network between containers also with *Docker*. Moreover, *Docker* also associates a container name to a DNS name into the virtual network, thus allowing for an easy network communication between containers.

As we wanted to test the architecture in an unstable networking context, we needed a tool to simulate errors on the virtual network. We chose *Pumba* (https://github.com/alexei-led/pumba), a network emulation tool for *Docker*. This open-source tool helps us simulate an unreliable network by letting us introduce lag, packet loss, or rate limitation into a virtual network between containers. Usage and setup of this tool are further described in Section 6.3.1.

Through the mentioned setup, we measured the performance of the following output parameters: network latency, bandwidth, CPU and RAM usage. The main focus of this architecture validation is the telemetry streaming from the drones to the server.

To measure latency, we use a timestamp contained in telemetries sent by the drones containers. Then, the telemetry is received by the server, which will log every telemetry received into a log file. This log contains the following information per telemetry received: drone ID, router ID, timestamp of the telemetry’s creation, timestamp of the telemetry’s reception by the server. Thus, we can measure the latency *l* as $l=T_{received}-T_{sent}$.

To measure bandwidth usage, we quantify the network transmission (*Tx*) and network reception (*Rx*) of each container composing the architecture.

In theory, we can calculate the following values, with *n* equal to the number of drones for one router, and *m* equal to the number of routers for a server:

$$Tx_{router\;j}=Rx_{router\;j}$$

$$Rx_{router\;j}=\sum_{i=1}^{n}Tx_{drone\;i}$$

$$Rx_{server}=\sum_{j=1}^{m}Tx_{router\;j}$$

We use random generated telemetry messages with an identical size to the “real” telemetries. Thus, we have an idea of the network consumption of the architecture.

While results may be biased by the multiple debug settings enabled on each program, they still give us a rough idea of the CPU/RAM usage of the architecture. These results will be particularly interesting for the drones and the server program. The former has to run on computers with a low energy consumption, thus a low CPU/RAM usage is mandatory for an effective usage on drones. Hence, we provide CPU/RAM usage for each component of the system—drone, router and server—when running the scenario with the highest load—namely, when drones are sending data at a 24 Hz update rate.

### 6.3. Configuration Settings

In the previous subsection, we described how we measure each performance parameter. In this subsection, we describe the different configuration settings we can make use of. We can modify the following settings:

- The data rate sent by drones
- The amount of drones
- The network’s quality

According to Calhoun et al. [20], the update should be the highest possible. More precisely, the following values should be used:

- 4 Hz as a minimum;
- 6 Hz or 10 Hz if resources are limited; and
- 24 Hz as an optimal value;

Therefore, we conducted our test with the following update rate values: 4 Hz, 10 Hz and 24 Hz.

As stated by Erdelj et al. [5], in an ideal case of continuous coverage of a Point of Interest, a user would need four drones running a scheduling algorithm. Thus, we simulated two fleets composed of four drones, i.e. four drones for one router, and two routers for one server.

#### 6.3.1. Configuration for Network Disruption Tests

As explained before, we can simulate a degraded network, which permits us to test situations closer to reality. The goal is not to realize a resilience test. Rather, we want to quantify how the architecture behaves in presence of an erratic network. Hence, we use the minimal configuration for testing, with a 4 Hz update rate.

We present the test configurations in Table 3.

**Figure 16:**
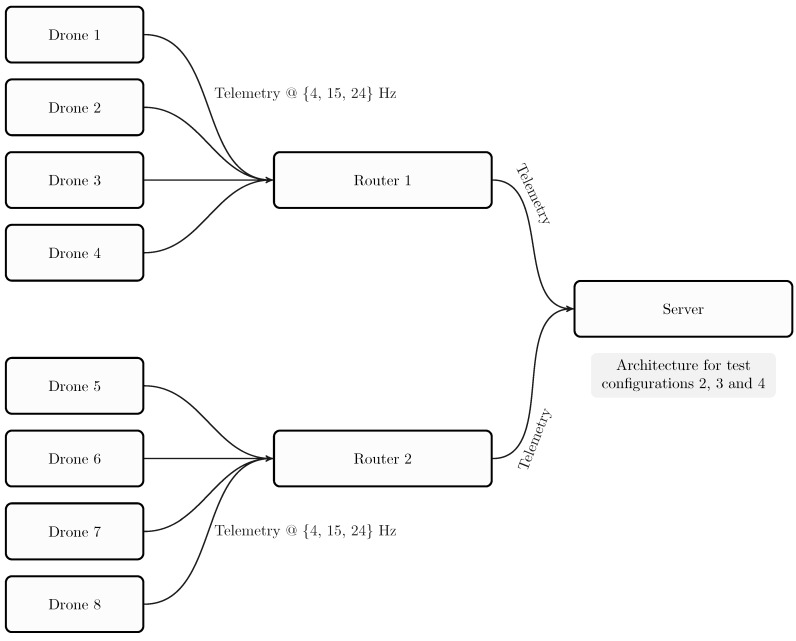
Test architecture for configurations.

Figure 16 presents the test architecture for all tests.

### 6.4. Results with No Network Disruption

In this section, we focus our attention on tests with no network disruption. The latter case is addressed in the next subsection.

Table 4 shows the results for each configuration. Latency is expressed in milliseconds.

The results with the *Delayed start* comment denotes that the high value of the max latency is due to a delayed start between the router and the drones. More precisely, if a router is down, the data sent by a drone are cached while the router is down, hence the high latency. A unusual high value of latency means that the telemetry was sent before the router was ready to operate. We can see that increasing the data rate to 24 Hz does not worsen significantly the latency. However, we ran these tests using a perfect virtual network, that is a network with no disruption; we compare these results with those obtained with an unstable network.

**Figure 17:**
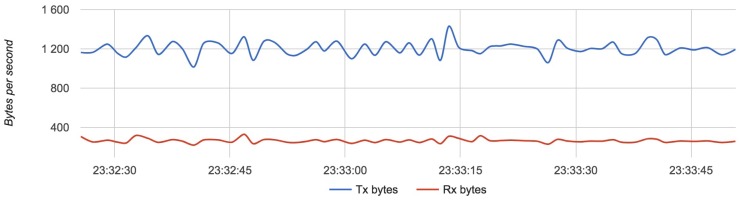
Bandwidth usage for a drone with Configuration 1.

**Figure 18:**
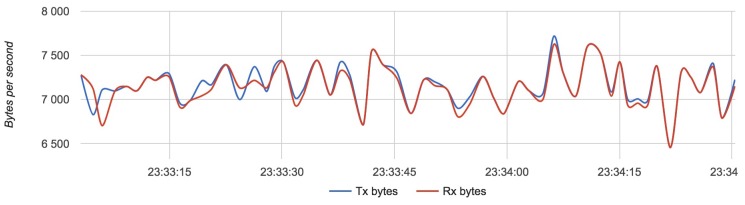
Bandwidth usage for a router with Configuration 1.

**Figure 19:**
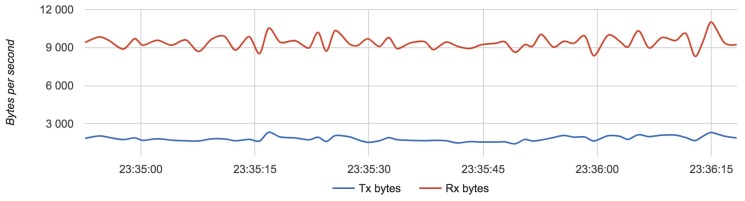
Bandwidth usage for a server with Configuration 1.

We compared the configuration with the lightest load (Configuration 1) with the configuration with the heaviest load (Configuration 3). Figure 17, Figure 18 and Figure 19 show the results for each component for Configuration 1.

The approximative value of bytes emitted by a drone is 1200 with an update rate of 4 Hz.

The approximate value of bytes received and transmitted by a router with four drones is 7250 with an update rate of 4 Hz.

The approximate value of bytes received by a server with two routers is 10,000 with an update rate of 4 Hz.

Results for Configuration 1 are summarized in Table 5.

**Figure 20:**
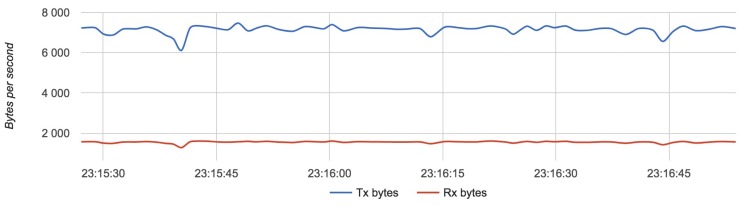
Bandwidth usage for a drone with Configuration 3.

**Figure 21:**
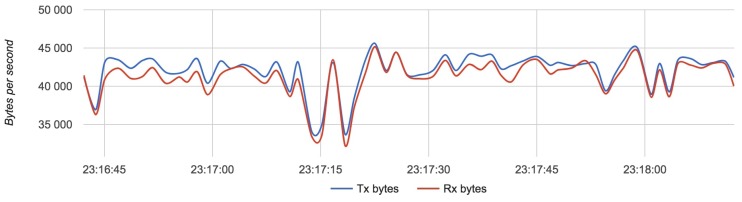
Bandwidth usage for a router with Configuration 3.

**Figure 22:**
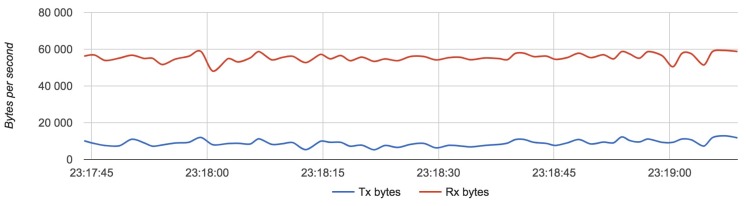
Bandwidth usage for a server with Configuration 3.

Figure 20, Figure 21 and Figure 22 show the results for each component in Configuration 3:

The approximate value of bytes emitted by a drone is 7000 with an update rate of 24 Hz.

The approximate value of bytes received and transmitted by a router with four drones is 43,000 with an update rate of 24 Hz.

The approximate value of bytes received by a server with two routers is 60,000 with an update rate of 24 Hz.

Results for Configuration 3 are summarized in Table 6.

We can note that the total number of data received by the server are multiplied by 6, which is equal to the amount we multiplied the data rate update by (from 4 Hz to 24 Hz).

The whole virtualized architecture was run on a MacBook Pro 13” late 2013 with the following technical specifications:

- CPU: 2.4 GHz i5-4258u
- RAM: 8 GB 1600 MHz DDR3
- HDD: 256 GB Solid State Drive

**Figure 23:**
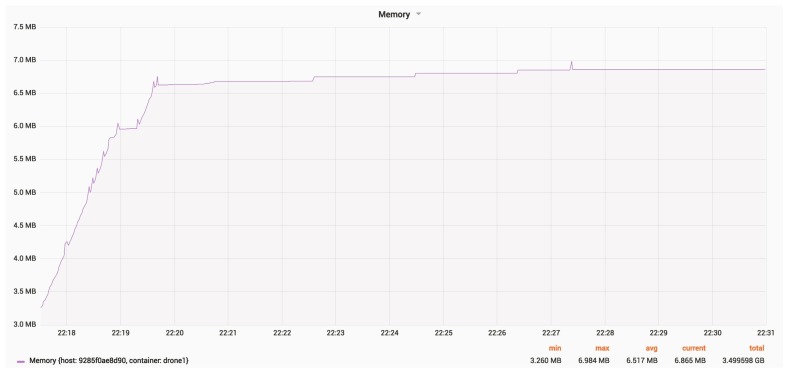
RAM usage for a drone.

**Figure 24:**
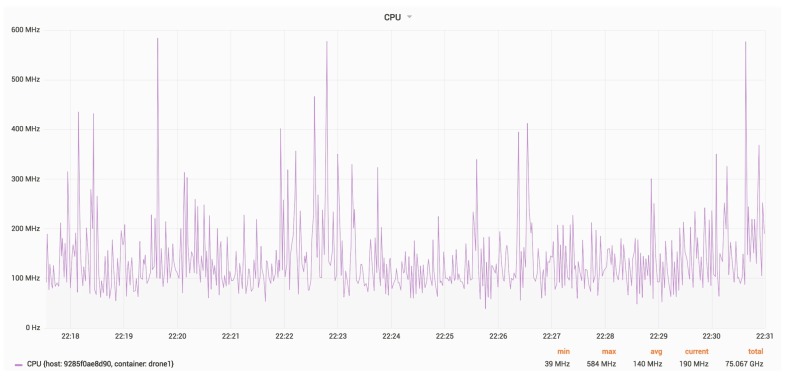
CPU usage for a drone.

Figure 23 and Figure 24 show the RAM and CPU usage for a drone, with an update rate of 24 Hz.

The average RAM usage is around 6.5 MB.

The average CPU usage is around 140 MHz.

**Figure 25:**
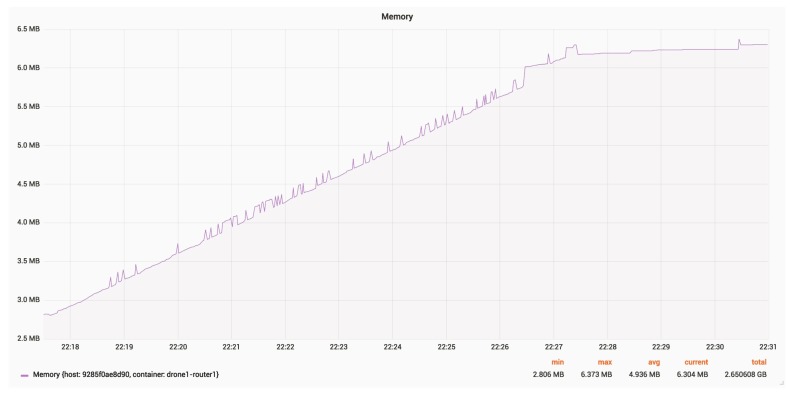
RAM usage for a router.

**Figure 26:**
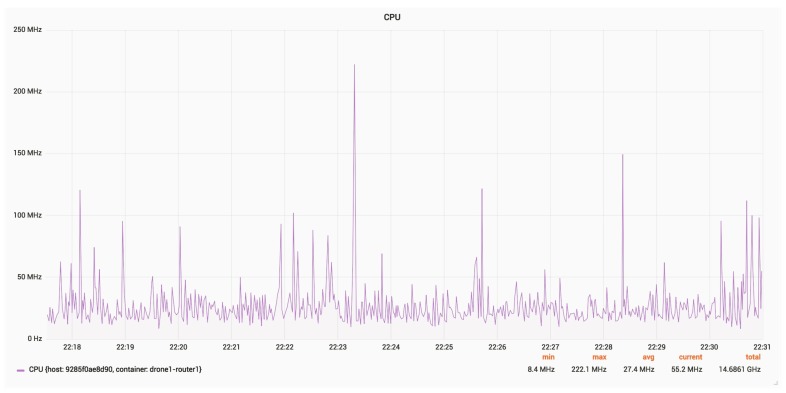
CPU usage for a router.

Figure 25 and Figure 26 present the RAM and CPU usage for a router, with an update rate of 24 Hz.

The average RAM usage is around 5 MB.

The average CPU usage is around 30 MHz.

**Figure 27:**
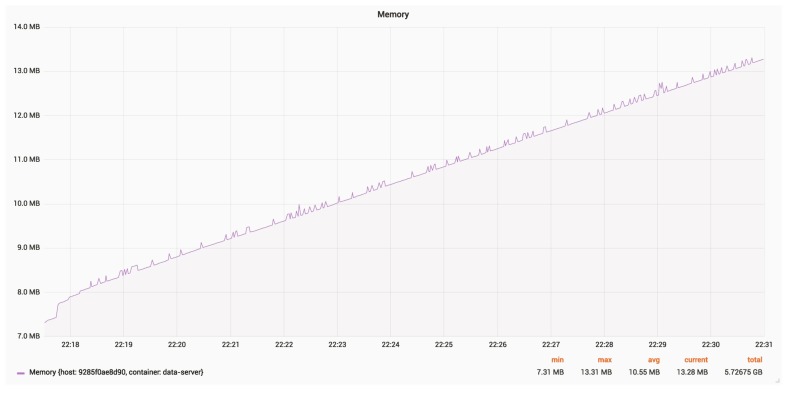
RAM usage for a server.

**Figure 28:**
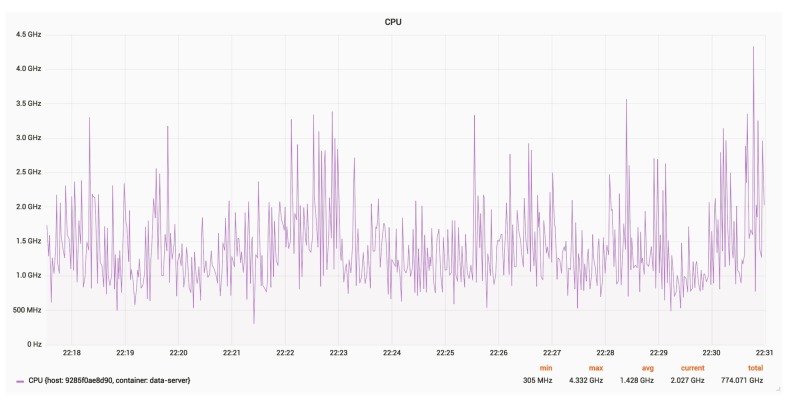
CPU usage for a server.

Figure 27 and Figure 28 show the CPU and RAM usage for a server, with an update rate of 24 Hz.

The average RAM usage is around 11 MB. The constantly growing memory usage may be due to debug logging.

The average CPU usage is around 1.430 GHz.

### 6.5. Results with Network Disruption

Using Configuration 1, where the drones send data at a 4 Hz rate, we tried to simulate a unreliable network with the following flaws:

- an added delay of 500±200 ms randomly distributed over the routers; and
- a 10% packet loss based on a Bernoulli independent model on the routers.

**Figure 29:**
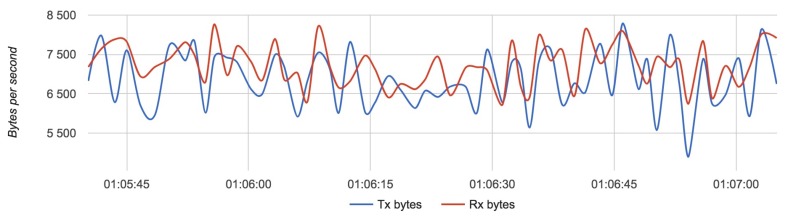
TX/RX difference with packet loss.

Figure 29 shows the packet loss simulated on a router.

**Figure 30:**
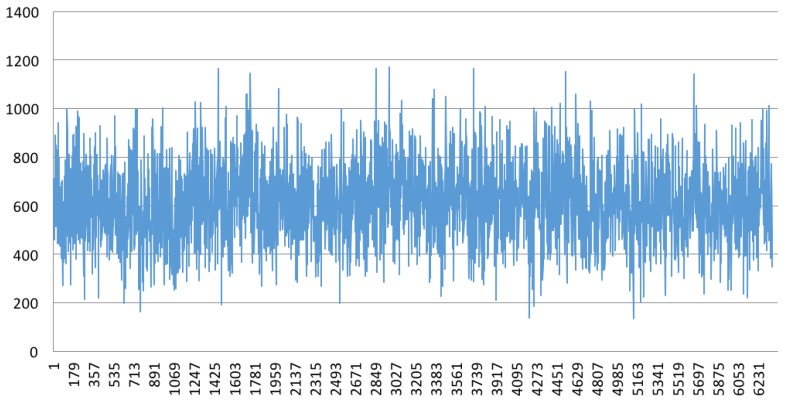
Latency when using an unstable network.

We also calculated the median latency: from 3 ms, we now have 616 ms. While this is mainly due to the hard 500 ms of latency added, it is still interesting to see how the architecture behaves when the network conditions are not stable. Figure 30 shows the latency for each received telemetry on a unstable network.

### 6.6. Drawbacks and Directions for Improvement

The main drawback of our software architecture lies into the server and its database, as there is only *one* server for *n* drones. A way to improve this is to transform the server and the database into a scalable *Docker* service, enabling multiple instances of the server to run. However, such scaling also gives rise to other issues, such as replication and inconsistency.

These issues are out of scope of this paper, but are tackled in the literature [16]. The other main drawback is represented by the quality of the network. Indeed, the latency mainly depends on the quality of the network rather than the number of data transmitted. Thus, to ensure the practical usage of the architecture, we first have to ensure a good quality of the network.

## 7. Conclusions

This paper introduces a novel architecture for disaster management, composed, for the first time, of: sensors, UAVs, and crowdsensing devices. The paper presents a detailed characterization of each component as well as an overview of the technologies useful for building a system architecture for data and video streaming with UAVs. It also details the design and implementation of such a system by properly selecting the right technology and characterizing the perceived and real information flows. The proposed architecture has been implemented in a first testbed as an output of two research projects, and has been validated and assessed for proper functioning and performance evaluation. The paper determines the main weak points of the architecture, and considers the possibility to implement it in a larger 5G IoT ecosystem by using virtualization.

## Figures and Tables

**Table 1:** Test results for Configuration 1: Video players in separate web clients.

Number of Clients	1	2	3	4	5	10
Latency (s)	0.27	0.28	0.25	0.26	0.42	1.41

**Table 2:** Test results for Configuration 2: Video players in the same web client.

Number of Clients	1	2	3	4	5	10
Latency (s)	0.24	0.28	0.27	0.39	0.53	2.3

**Table 3:** Test configurations.

Configuration	Data Rate	Drones per Router	Routers	Notes
1	4 Hz	4	2	Minimal fleet
2	10 Hz	4	2	Medium rate fleet
3	24 Hz	4	2	High rate fleet
1b	4 Hz	4	2	Minimal unstable network
3b	24 Hz	4	2	High unstable network

**Table 4:** Bandwidth usage.

Configuration	Data Rate	Median Latency	Max Latency	Min Latency	Notes
1	4 Hz	3 ms	460 ms	0 ms	Delayed start
2	10 Hz	3 ms	6152 ms	0 ms	Delayed start
3	24 Hz	4 ms	1016 ms	0 ms	No delayed start

**Table 5:** Bandwidth usage for Configuration 1.

Component	Usage	Notes
Drone	1200 bytes	TX Only
Router	7250 bytes	TX & RX
Server	10,000 bytes	RX Only

**Table 6:** Bandwidth usage for Configuration 3.

Component	Usage	Notes
Drone	7000 bytes	TX Only
Router	43,000 bytes	TX & RX
Server	60,000 bytes	RX Only
